# EFFECTS OF MUSCLE ALTERATIONS AND REHABILITATION ON MOBILITY IN CHRONIC CONDITIONS OF AGING

**DOI:** 10.1093/geroni/igz038.2125

**Published:** 2019-11-08

**Authors:** Monica C Serra

**Affiliations:** Emory University, Atlanta, Georgia, United States

## Abstract

Age-related changes in muscle morphology and composition are associated with the development of mobility impairments, an increased risk for institutionalization, and mortality. While primary aging may account for some of these changes, the presence of aging related co-morbid conditions such as frailty, stroke, diabetes, and Parkinson’s disease is associated with muscle fiber atrophy, increased intramuscular fat, changes in muscle fiber distribution, and vascular dysfunction that occurs in both large and small vessels. These changes ultimately result in impaired substrate delivery to the muscle, reduced capacity for muscle regeneration, and anabolic resistance leading to declines in balance, strength, and endurance. It is important to understand the biological underpinnings of the changes in muscle following each of these conditions to develop effective preventive measures and maximize restorative rehabilitation protocols. This session will discuss the changes in muscle from the fiber level (i.e., changes in innervation and muscle fiber type proportion) to the systemic level (i.e., changes in specific postural and locomotor muscles), and their relationship to mobility, balance, and function. We will describe techniques (i.e., imaging, EMG, and muscle biopsies) used to assess muscle changes and their applicability in rehabilitation research. Further, we will discuss clinical recommendations for exercise to preserve and enhance skeletal muscle in the aging adult.

